# Evaluation of Microclimatic Detection by a Wireless Sensor Network in Forest Ecosystems

**DOI:** 10.1038/s41598-018-34832-7

**Published:** 2018-11-06

**Authors:** Jiaxin Jin, Ying Wang, Hong Jiang, Xiaofeng Chen

**Affiliations:** 1 School of Earth Sciences and Engineering, Hohai University, Nanjing, 211100 China; 2 College of Culture Industry and Tourism Management, Sanjiang University, Nanjing, 210012 China; 3 International Institute for Earth System Science, Nanjing University, Nanjing, 210023 China; 4 Beijing Pri-eco Technology Co., LTD, Beijing, 100000 China

## Abstract

Timely and accurate detection of microclimates is extremely valuable for monitoring and stimulating exchanges of mass and energy in forest ecosystems under climate change. Recently, the rapid growth of wireless sensor networks (WSNs) has provided a new approach for detecting microclimates in a complex environment at multiple temporal and spatial scales. However, applications of wireless sensors in forest microclimate monitoring have rarely been studied, and the corresponding observation accuracy, error sources and correction methods are not well understood. In this study, through field experiments in two typical subtropical forest ecosystems in Zhejiang Province, China, the accuracy of the temperature and humidity observed by the wireless sensors was evaluated against standard meteorological data. Furthermore, the observation error sources were analyzed and corresponding correction models were established. The results showed that the wireless sensor-based temperature and humidity values performed well within the total observation accuracy. However, the observation errors varied with season, daily periodicity and weather conditions. For temperature, the wireless sensor observations were overestimated during the daytime while they were underestimated during the nighttime. For humidity, the data observed by the wireless sensors generally appeared as overestimates. Adopting humidity as the corrected factor, correction models were established and effectively improved the accuracy of the microclimatic data observed by the wireless sensors. Notably, our error analysis demonstrated that the observation errors may be associated with the shell material of the wireless sensor, suggesting that shading measures for the wireless sensors should be considered for outdoor work.

## Introduction

Climate warming, variabilities in precipitation intensity and frequency, and resulting climatic events (e.g., extreme drought and flood) have already significantly influenced the physiological and ecological processes of forest ecosystems^1–3^. The exact and timely acquisition of forest meteorological information, especially on forest microclimates, is very valuable for monitoring and investigating carbon and water cycles, energy balance and interactions between the canopy and ground within forest ecosystems under climate change^4,5^.

Currently, the main means of monitoring forest microclimates is through *in situ* observations using a thermometer screen or auto weather station. This approach usually ensures the observational accuracy of microclimatic variables. However, it is too expensive and requires too much labor to meet the need for continuous time monitoring over a relatively wide region due to limitations in power supply, etc. More importantly, it is infeasible to place the sensors in a complex terrain or at multiple layers and to adjust the layout regularly based on demand and supply^6^. Although the spatial pattern of the microclimates (e.g., temperature) of the canopy or land surface could be derived from remote sensing data, large uncertainties may be associated with the spatial and spectral resolution of the satellite^7^. Similarly, successive observations cannot be achieved due to the limitations in satellite transit time.

With the developments of wireless sensor network (WSN) technology, also known as the Internet of Things (IOT), wireless sensors have been increasingly used in monitoring the ecological environment^8–17^. Wireless sensors are characterized by their low costs, high monitoring accuracy, flexible layouts and high-frequency continuous data^18,19^. These advantages make detecting microclimatic variables in a complex ecosystem (e.g., forest ecosystem) possible^20,21^. Hence, wireless sensors could provide valuable data for investigating and simulating carbon and water cycles and energy balance within an ecosystem^6,22–24^. Although applications of wireless sensors for forest microclimate monitoring and the corresponding evaluations have been explored^4,25^, the observational accuracy of the sensors, sources of observational bias and correction approaches are less understood^26–28^.

To fill these gaps, this study investigated the suitability of wireless sensors for forest microclimate monitoring. Two typical subtropical forest sites, a mixed broadleaf forest site and bamboo forest site, were chosen as the study sites. Compared to observations from standard meteorological instruments in the study sites, the accuracies of the temperature and humidity data observed by the wireless sensors and the error sources were detected. Then, correction models of the climatic variables were established to improve the monitoring effectiveness of the wireless sensors in forest microclimates. Achieving these goals is beneficial to guiding sensor placement in forest ecosystem and provide a scientific basis for improving the designation and data correction of wireless sensors.

## Results and Discussion

### Overall observation accuracies

**Figure 1:**
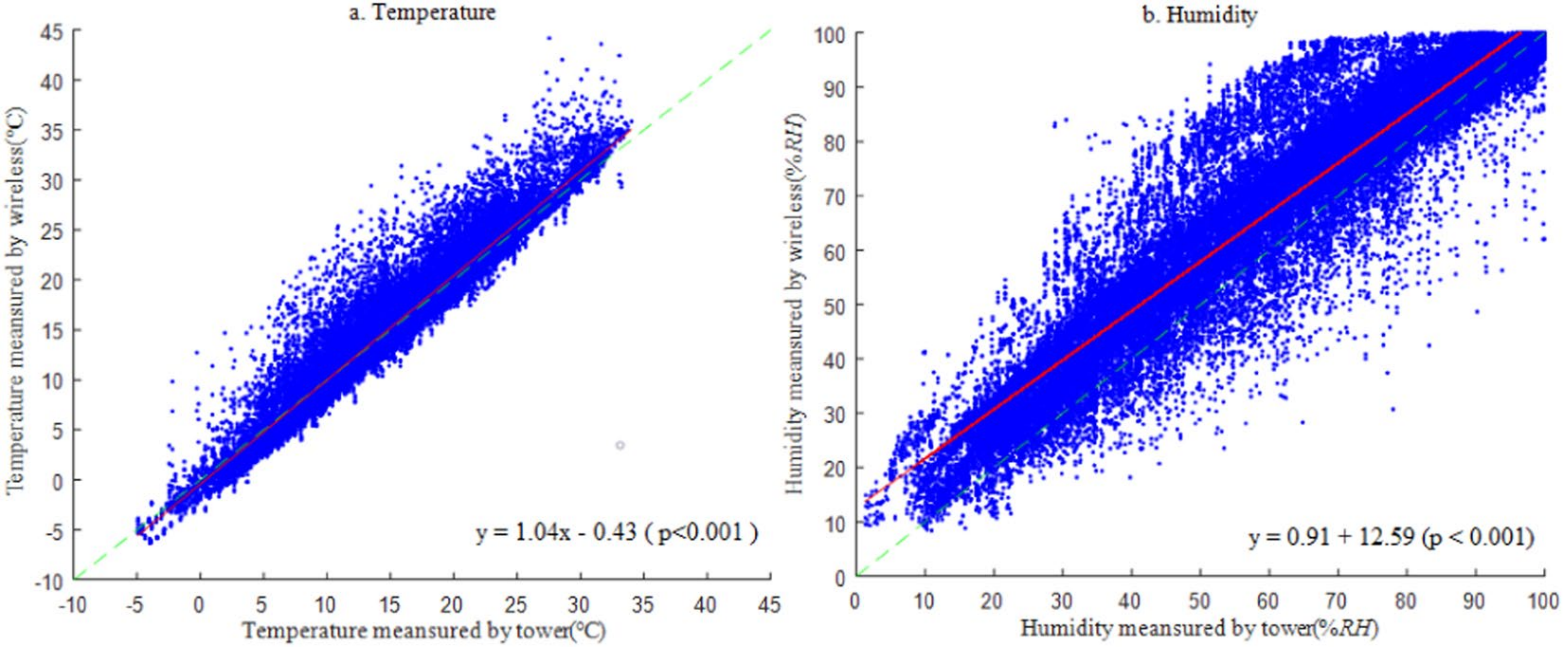
Overall accuracy evaluation of temperature (**a**) and humidity (**b**) observed by the wireless sensor. (n = 39796 and 44227 points for temperature and humidity, respectively).

First, the overall accuracy of the temperature and humidity observed by the wireless sensors were evaluated. All the wireless sensor-based observations were collected, and the outliers (out of three standard deviations) were removed. The valid data were compared to the corresponding standard meteorological observations at both the mixed broadleaf forest site and bamboo forest site, as shown in Fig. 1. The results showed that the wireless sensors generally performed well in terms of observation accuracy with slight overestimates. The overall average temperature and humidity observed by the meteorological gradient systems were 14.9 °C and 60.2% RH, respectively, and the overall average temperature and humidity observed by the wireless sensors were 15.1 °C and 67.1% RH, respectively. The linear regression analysis showed that for temperature, the slope and R^2^ of the regression between the gradient and sensor observations were 1.04 and 0.94, respectively, and the Root Mean Square Error (RMSE) and Evaluation Accuracy (EA) were 1.94 and 87% (Fig. 1a). For humidity, the slope, R^2^, RMSE and EA were 0.91, 0.89, 10.9 and 82%, respectively (Fig. 1b).

### Diurnal and seasonal observation accuracies

**Figure 2:**
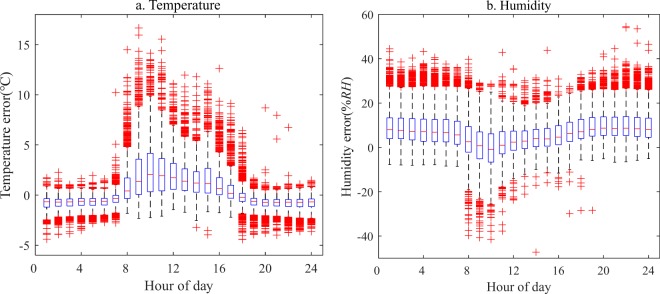
Diurnal variations in the errors of temperature (**a**) and humidity (**b**) observed by the wireless sensors. The x-axis indicates the time of day, and the y-axis indicates the observation errors of the wireless sensors. The upper and lower boundaries of the box indicate the 75th and 25th percentiles, respectively, (referred as Q3 and Q1, respectively) of the error bound, and the red line within the box marks the median. The error bars above and below the box indicate the maximum (Q3 + 1.5*IQR, where IQR = Q3 − Q1) and minimum (Q1 − 1.5*IQR), respectively. The red crosses indicate outlying points.

Diurnal variabilities of errors of temperature and humidity observed by the wireless sensor are shown in Fig. 2. The errors, which were the differences between the wireless sensor observations and corresponding gradient observations, were summarized by hour of day. A positive error indicates the wireless sensor exhibited an overestimate of the standard value. The results showed that for temperature, the errors mainly appeared during the daytime (7:00 am - 5:00 pm), accounting for 64% of the total errors. The average diurnal error was 1.6 °C, and the maximum value of errors appeared at 10:00 am-11:00 am with an average of 2.7 °C. During the nighttime (6:00 pm - 6:00 am), the errors were mainly negative and relatively small (average of 0.7 °C) compared to those during the daytime. In general, humidity was overestimated by the wireless sensors throughout the entire day. Positive errors mainly occurred during the nighttime with an average of 8.7% RH, which accounted for 74% of the total humidity errors. The average error during the daytime was 4.3% RH, and the minimum errors appeared at 9:00 am.

**Figure 3:**
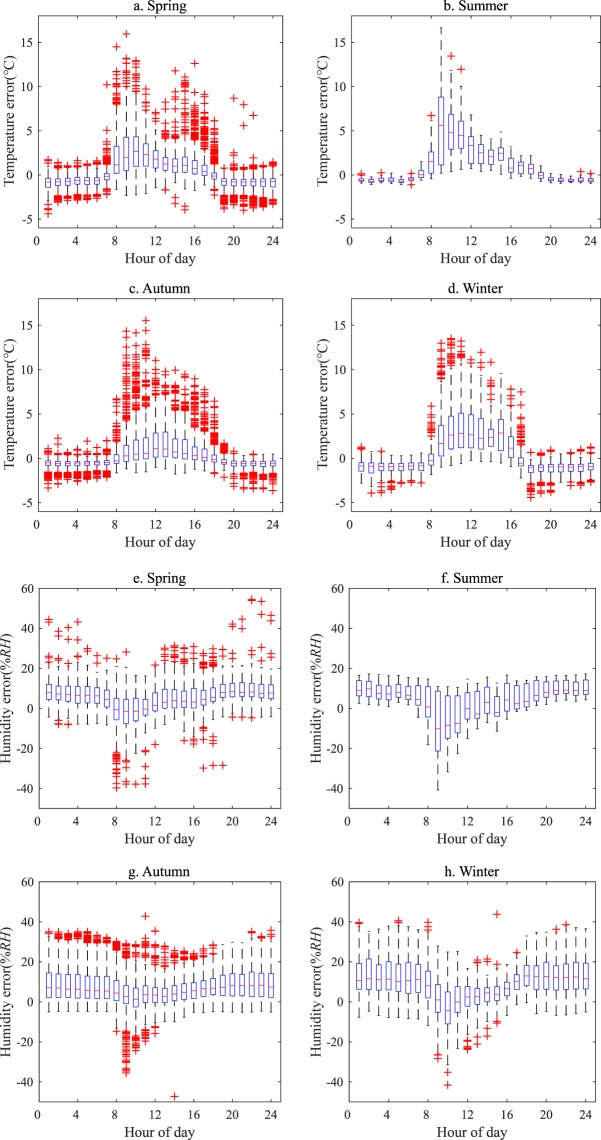
Diurnal variations in the errors in temperature (**a**–**d**) and humidity (**e**–**h**) observed by the wireless sensors in the spring, summer, autumn and winter. The symbols in this figure are the same as those in Fig. 2.

The seasonal variations in the errors of temperature and humidity observed by the wireless sensor were detected, shown in Fig. 3. Like the diurnal variabilities in the errors throughout the year, the temperature errors were positive and mainly appeared in the daytime of each season, while they were negative at night with fewer fluctuations. The daytime errors (average value) accounted for 64% (1.6 °C), 82% (2.7 °C), 62% (1.1 °C) and 64% (1.9 °C) of the total errors in each season, respectively. The maximum value of errors was exhibited in the summer. The average errors in the nighttime temperatures were −0.8 °C, −0.5 °C, −0.6 °C and −1 °C, respectively, and the maximum value of errors appeared in the winter. For humidity, the errors mainly were positive and larger during the nighttime. The nighttime errors accounted for 73%, 73%, 70% and 73% of the total humidity errors in each season, respectively. The nighttime (daytime) errors were 7.7% RH (2.8% RH), 8.9% RH (0.36% RH), 9.3% RH (4.9% RH) and 12.9% RH (4.6% RH), respectively. The results demonstrated that the observation errors in the wireless sensors were impacted by variations in not only diurnal but also seasonal climates to some extent, especially by daytime high temperatures in the summer and nighttime low temperatures in the winter.

### Observation accuracy in different weather

In addition to the diurnal variations in meteorology, the variations in air humidity with weather conditions, such as cloudy and rainy, may lead to some random errors observed by the wireless sensor. Here, we further analyzed the diurnal differences between the wireless sensors and gradient system observations in clear and cloudy/rainy conditions. The cloudy/rainy condition was defined as a 5-hour duration in which the humidity was over 80% RH.

**Figure 4:**
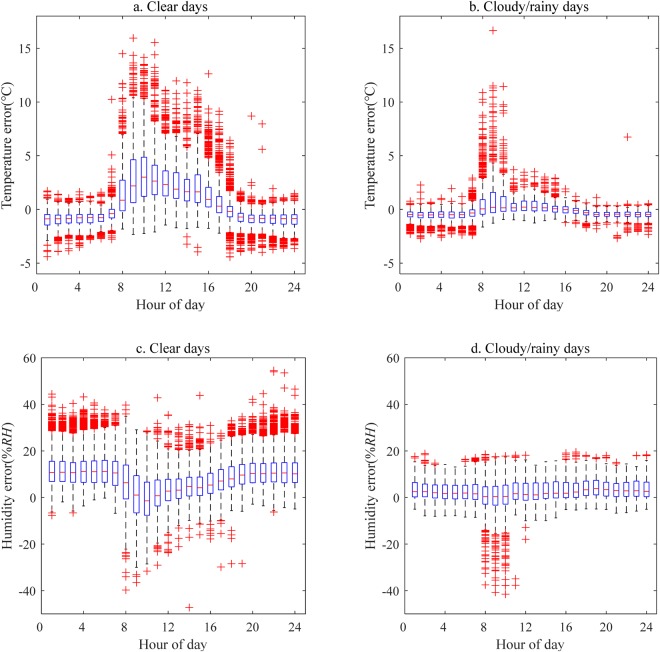
Diurnal variations in the errors in temperature (**a**,**b**) and humidity (**c**,**d**) observed by wireless sensors on clear and cloudy/rainy days. The symbols in the figure are the same as those in Fig. 2.

The results (Fig. 4) showed that the observation errors were larger in clear rather than cloudy/rainy conditions. In clear conditions, the diurnal errors in temperature accounted for 67% of the total errors, and the average was 2 °C, while the average of the nighttime errors was −0.9 °C. On cloudy/rainy days, there was not an obvious difference between the diurnal and nighttime errors, and the average of these errors was −0.3 °C and −0.5 °C, respectively. For humidity, the nighttime errors accounted for 84% of the total errors in clear conditions, and the average error during the nighttime and daytime was 12% RH and 4.3% RH, respectively. Like with temperature, the difference between the diurnal and nighttime errors was also not significant in cloudy/rainy conditions. The average of the diurnal and nighttime errors in humidity was 2.1% RH and 3.1% RH, respectively.

In summary, the temperature and humidity errors in the wireless sensors were affected by variabilities in temperature and humidity with day and night, seasons and weather. The error characteristics implied that the observation errors may be associated with the sensor shell. The shell used in this study is plastic, and the sensor is wrapped in the shell. During a clear day, the shell was rapidly warmed by solar radiation, leading to a higher temperature in the shell than that of the ambient air and consequently an overestimate of the temperature. Nevertheless, during a clear night, a weak atmospheric counter radiation caused a low temperature and a corresponding low saturated vapor pressure^29^. The plastic was more able to dissipate heat, resulting in a lower temperature and more dew in the shell. Hence, the wireless sensor may underestimate air temperature and overestimate air humidity.

### Corrections of the wireless sensor observations

According to the evaluations mentioned above, the errors in temperature and humidity observed by the wireless sensors may be related to the air humidity within the diurnal period, which partly resulted from the weather and the sensor in the shell. Correlations (r) between errors and the corresponding humidity observed by the gradient system were detected during the daytime and nighttime. The results showed that for temperature the daytime errors significantly and negatively related to humidity (r = −0.66, p < 0.001), while the nighttime errors showed a significant positive correlation with humidity (r = 0.75, p < 0.001). In contrast, for humidity, the daytime errors showed a significant positive relationship with humidity (r = 0.57, p < 0.001), while the nighttime errors exhibited a significant negative correlation with humidity (r = −0.67, p < 0.001). Hence, to correct the observation error in the wireless sensor, humidity was selected as the correction factor, and the error correction model of the wireless sensor was established by hour based on the linear regression function.

The relationships between the observation errors in the wireless sensors temperature and humidity are shown as follow:

1 $${\rm{\Delta }}{\rm{T}}={{\rm{k}}}_{{\rm{i}}}\times {\rm{RH}}\mbox{'}+{{\rm{b}}}_{{\rm{i}}},$$

2 $${\rm{\Delta }}\mathrm{RH}={{\rm{k}}}_{{\rm{i}}}\times {\rm{RH}}\mbox{'}+{{\rm{b}}}_{{\rm{i}}}$$

where i (i = 1~24) indicates the hour of the day. ΔT and ΔRH indicate temperature and humidity errors observed by the wireless sensors at a certain hour, respectively, and RH’ indicates the corresponding relative humidity. k_i_ and b_i_ indicate the slope and interception of linear regression at the hour, respectively.

**Figure 5:**
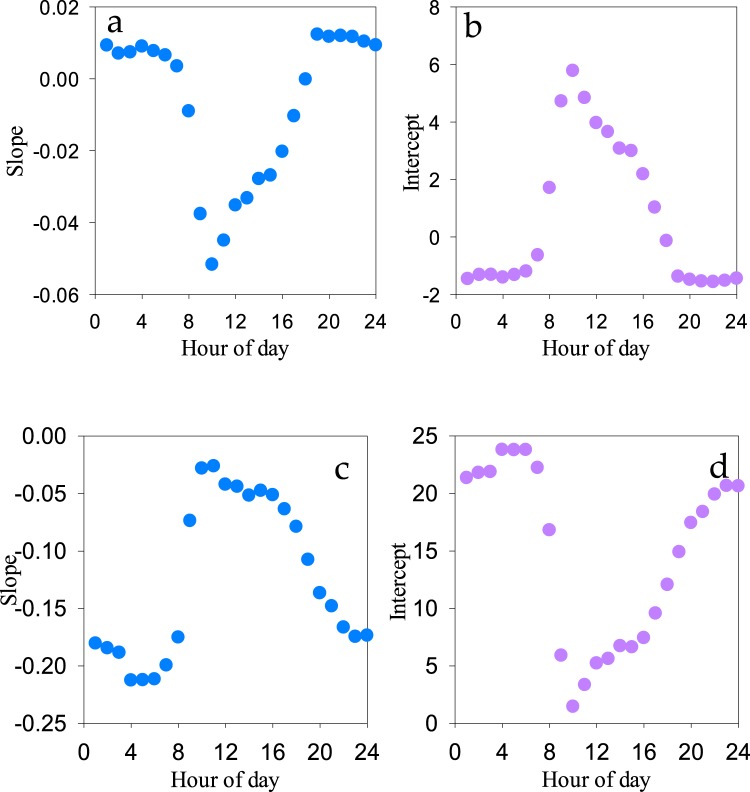
The slope and intercept of the linear regression of the wireless sensor-based temperature (**a**,**b**) and humidity (**c**,**d**) errors with air humidity in a diurnal cycle. The x-axis indicates the time of day (i.e., hours). The y-axis indicates the regression parameters.

The parameters of the correction models are shown in Fig. 5. For temperature, k exhibited an inverted U-shape in the diurnal periodicity, while for humidity, k appeared in an opposite pattern. For temperature, the values of k in the nighttime were positive with an average of 0.009, while those in the daytime were negative with an average of −0.02. For humidity, the regression slopes were larger in absolute terms during the nighttime than those during the daytime, and the averages of the slopes were −0.18 and −0.05, respectively. Based on the relationships between the observation errors and humidity, the correction models of temperature (T) and humidity (RH) were established as follows:

3 $${\rm{T}}={{\rm{T}}}_{{\rm{raw}}}-{\rm{\Delta }}{\rm{T}}={{\rm{T}}}_{{\rm{raw}}}-({{\rm{k}}}_{{\rm{i}}}\times {\rm{RH}}\mbox{'}+{{\rm{b}}}_{{\rm{i}}}),$$

4 $${\rm{RH}}={{\rm{RH}}}_{{\rm{raw}}}-{\rm{\Delta }}\mathrm{RH}={{\rm{RH}}}_{{\rm{raw}}}-({{\rm{k}}}_{{\rm{i}}}\times \mathrm{RH}\mbox{'}+{{\rm{b}}}_{{\rm{i}}})$$

The corrected T (RH) was the difference between the raw temperature (humidity) observed by the sensors (T_raw_ (RH_raw_)) and ΔT (ΔRH), which was calculated considering formula 3 (4).

**Figure 6:**
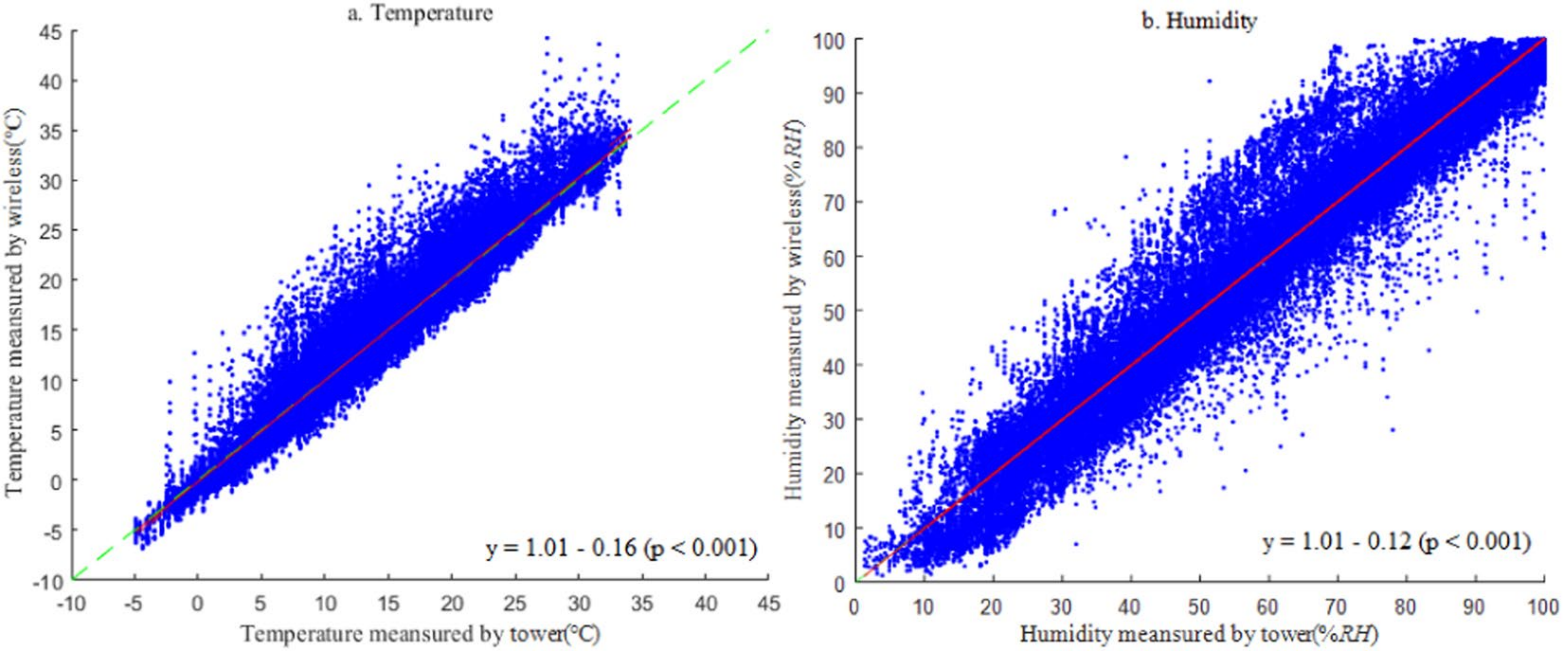
Accuracy evaluation of the calibrated temperature (**a**) and humidity (**b**) observed by the wireless sensors.

After the correction, the temperature and humidity observed by the wireless sensors obviously improved and were more consistent with the standard values. The evaluation results (Fig. 6) showed that the slope (k) and R^2^ of the linear regression between the wireless sensor-based and standard temperature were 1.01 and 0.96, respectively, and the RMSE and EA were 1.35 °C and 91%, respectively. For humidity, the k and R^2^ between the calibrated sensor-based and standard observations were 1.002 and 0.93, respectively, and the RMSE and EA were 7.1% RH and 88%, respectively.

## Conclusions

Based on field experiments, this study evaluated the accuracy of the temperature and humidity observed by wireless sensors in two typical subtropical forest ecosystems and further determined the sources of the observation error and established the corresponding correction models. Our findings showed that the sensor-based temperature and humidity performed well in terms of the total observation accuracy, which is suitable for monitoring forest microclimates. However, the observation errors varied with seasons, day and night, as well as weather conditions. For temperature, the wireless sensor observations exhibited an overestimate during the daytime, while it showed an underestimate for temperature during the nighttime. Moreover, the error was larger during the daytime than during the nighttime, and the error was larger on clear days than on cloudy/rainy days. For humidity, the data observed by the wireless sensors generally appeared as an overestimate, and the observed errors were larger at night and on clear days than those during the daytime and on cloudy days. Adopting humidity as the corrected factor, the correction models were established at an hour scale and were shown to effectively improve data accuracy and the applicability of the wireless sensors in forest ecosystems. Notably, the error analysis demonstrated that the sensor-based observation errors in temperature and humidity may be associated with the shell material of the wireless sensor. Hence, we suggest that shading measures should be considered when wireless sensors are arranged in the open air in future work or use other heat spreader materials as opposed to plastics in the shell of the sensor.

## Methods

### Study sites

In this study, two typical subtropical forest flux-observation sites in Zhejiang Province, China were selected as the study sites. One study site is the Tianmushan forest ecosystem research site in the Chinese Forest Ecosystem Research Network (CFERN) in Hangzhou, which represents an evergreen and deciduous mixed broadleaf forest ecosystem^30^. This site is located in Tianmushan National Nature Reserve, where the annual temperature ranges from 8.8 °C to 14.8 °C, annual precipitation ranges from 1390 mm to 1870 mm and relative humidity ranges between 76% and 81%. A 40-meter-high flux observation tower was established in the core area of the reserve (30°20′59″N, 119°26′13″E). The other study site is the flux-observation site in the moso bamboo (Phyllostachys pubescens) technology park in Anji, which represents the Mao bamboo forest ecosystem^31^. Like the other study site, a 40-meter-high tower stands in the park (30°28′34″N, 119°40′25″E), where the annual temperature, precipitation and relative humidity are approximately 16.6 °C, 1270 mm and over 70%, respectively.

The meteorological gradient observation system (hereafter referred as the gradient system) on the two flux towers were similar, including wind speedometers, thermometers and moisture meters placed at 2 m, 7 m, 11 m, 17 m, 23 m, 30 m and 38 m along each tower. These gradient system observations were treated as the standard meteorological observations with high observation accuracy. Additionally, two infrared temperature instruments (SI-1111) were placed at 2 m and 23 m along each tower to capture the land surface and canopy temperatures, respectively. The meteorological information was automatically collected every 30 minutes.

### Wireless sensor network

The wireless sensor used in this study is the Telos-Rev.B sensor node, which loads the temperature/humidity sensor module SHT11^32^. The measurement range for temperature is from −40 °C to 123.8 °C, and the accuracy of the measurement is ±0.3 °C at a 0.01 °C resolution. The measurement range for humidity is from 0 to 100% (RH), and the observational accuracy is ±3.0% (RH) at a 0.01% (RH) measurement resolution.

In this study, the wireless sensor network was based on the GreenOrbs deployments^33^. Three types of nodes were implemented in this study, including wireless sensor nodes equipped with the temperature/humidity sensor (abbreviated to sensor nodes), sink notes and task manager node. The information was collected by each sensor node and then transferred to sink notes through adjacent routers. Finally, the information was transferred to the task manager nodes by the bridging ability of the sink nodes, so that it could be handled in the user terminal.

### Field experiments

**Figure 7:**
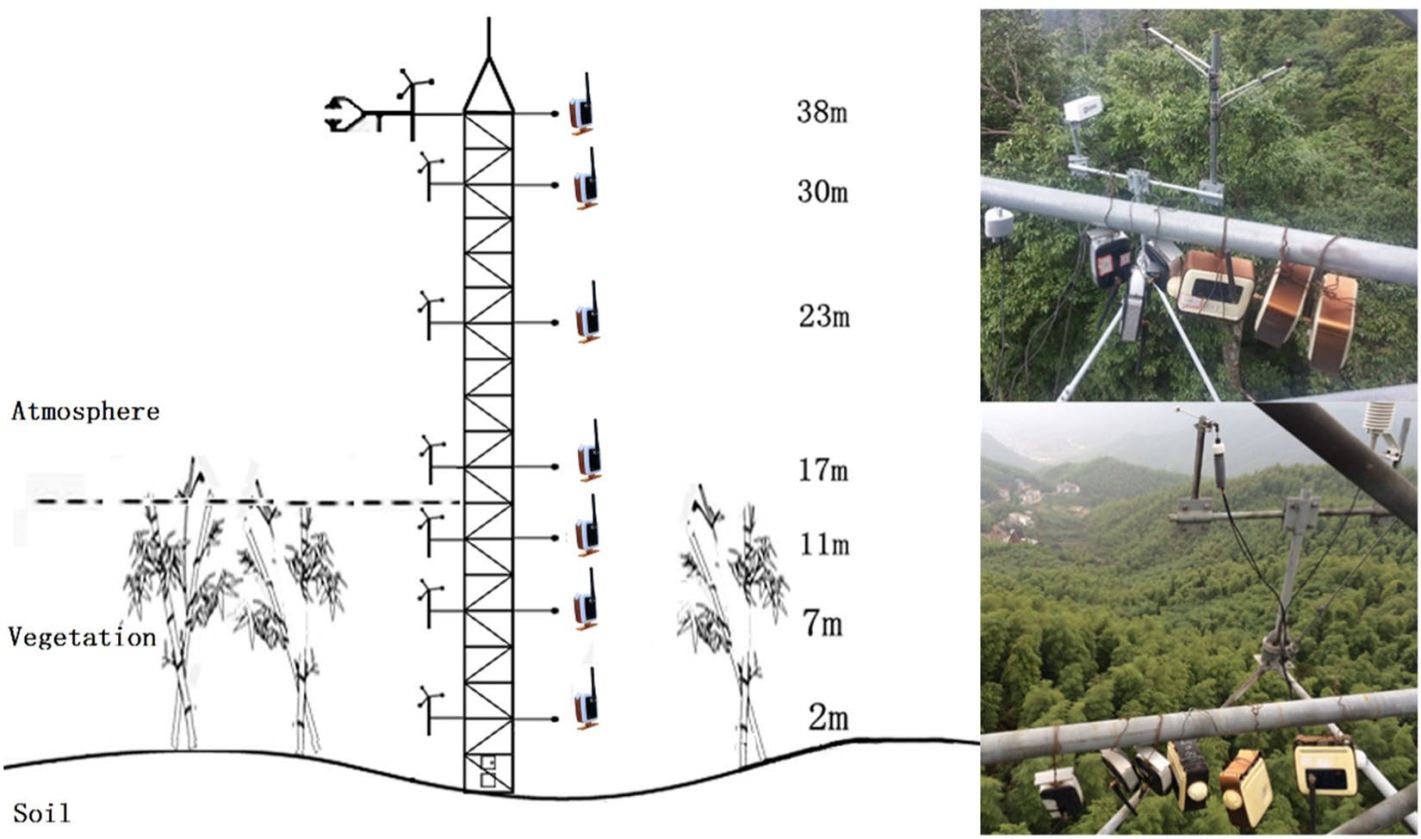
Layout of the wireless sensors on the flux towers.

The wireless sensors were placed along the flux tower at the Tianmushan and Anji sites, and the accuracies were evaluated by the corresponding gradient system observations. The details of the experiments are described as follows. Two or more wireless sensors were placed at each layer of the flux towers and placed as close as possible to the instruments of the gradient system to reduce observation uncertainties. The layouts of the wireless sensors on the flux towers are shown in Fig. 7.

Each field experiment generally lasted 10~20 days due to the limitation of the battery life of the wireless sensor. In total, seven and ten field experiments were carried out in the Tianmushan site from September 2013 to May 2015 and Anji site from December 2011 to October 2012, respectively. Excluding the missing data due to power or sensor failures and severe weather, there were 97 site-days of valid data samples (Table 1). The sensor-based temperature and humidity data essentially covered all seasons and different weather types (e.g., clear, cloudy and rainy day), providing robust temporal coverage and reliability.

**Table 1:** The dates of the wireless sensor observations in this study.

Study site	Start date	End date	number of days
Tianmushan site	2013/9/1	2013/9/21	21
	2013/11/4	2013/11/29	6
	2014/3/21	2014/4/10	21
	2015/5/13	2015/5/22	10
Anji site	2011/12/6	2011/12/23	18
	2012/6/15	2012/6/18	4
	2012/7/4	2012/7/9	6
	2012/10/17	2012/10/26	10

Considering the battery life and data integrity, the sampling period of the wireless sensor was set to 10 minutes, and the data within a half hour were averaged. The data outliers with network-transmission problems were discarded. Additionally, based on the long-term microclimatic observations of the gradient systems the reasonable ranges of temperature and humidity in the study sites were 15 °C~45 °C and 0~100%, respectively. Hence, the wireless sensor observations beyond these ranges were excluded in further analyses.

### Statistical analysis

For the accuracy evaluation of the wireless sensors, the linear regression relationships between the wireless sensor- and flux tower-based climatic observations were detected in our present work. Additionally, root mean square error (RMSE) and evaluation accuracy (EA) were also used, which are shown as the following:

5 $${\rm{RMSE}}=\sqrt{\frac{{\sum }_{i=1}^{n}{({\rm{\Delta }}Sensor-{\rm{\Delta }}Tower)}^{2}}{n},}$$

6 $${\rm{EA}}=(1-\frac{RMSE}{Mean})\times 100,$$

where ΔSensor and ΔTower represent the wireless sensor- and flux tower-based climatic observations, respectively, n represents the sample size, mean denotes the average of the observations from the gradient system or profile system.

In this study, the overall accuracies of the temperature and humidity observed by the wireless sensors were first evaluated. Considering the potential impacts of climatic conditions and plant seasonal activities on equipment precision, observational accuracy of the wireless sensors were further detected during different seasons, at different daily periodicities and in different weather conditions. Meanwhile, the error characteristics and causes were analyzed. Then, an optimized correction factor was selected to establish correction models for adjusting the original sensor-based observations. Finally, the data before and after the correction were compared.
